# Differences in vasomotor function of mesenteric arteries between Ossabaw minipigs with predisposition to metabolic syndrome and Göttingen minipigs

**DOI:** 10.1152/ajpheart.00719.2023

**Published:** 2023-12-22

**Authors:** Chantal Eickelmann, Helmut Raphael Lieder, Michael Sturek, Gerd Heusch, Petra Kleinbongard

**Affiliations:** ^1^Institute for Pathophysiology, West German Heart and Vascular Center, University of Essen Medical School, Essen, Germany; ^2^CorVus Biomedical, LLC, and CorVus Foundation, Inc., Crawfordsville, Indiana, United States

**Keywords:** genetic predisposition, Ossabaw minipig, vasomotor function

## Abstract

Metabolic syndrome predisposes and contributes to the development and progression of atherosclerosis. The minipig strain “Ossabaw” is characterized by a predisposition to develop metabolic syndrome. We compared vasomotor function in Ossabaw minipigs before they developed their diseased phenotype to that of Göttingen minipigs without such genetic predisposition. Mesenteric arteries of adult Ossabaw and Göttingen minipigs were dissected postmortem and mounted on a myograph for isometric force measurements. Maximal vasoconstriction to potassium chloride (KCl_max_) was induced. Cumulative concentration-response curves were determined in response to norepinephrine. Endothelium-dependent (with carbachol) and endothelium-independent (with nitroprusside) vasodilation were analyzed after preconstriction by norepinephrine. In a bioinformatic analysis, variants/altered base pairs within genes associated with cardiovascular disease were analyzed. KCl_max_ was similar between the minipig strains (15.6 ± 6.7 vs. 14.1 ± 3.4 ΔmN). Vasoconstriction in response to norepinephrine was more pronounced in Ossabaw than in Göttingen minipigs (increase of force to 143 ± 48 vs. 108 ± 38% of KCl_max_). Endothelium-dependent and endothelium-independent vasodilation were less pronounced in Ossabaw than in Göttingen minipigs (decrease of force to 46.4 ± 29.6 vs. 16.0 ± 18.4% and to 36.7 ± 25.2 vs. 2.3 ± 3.7% of norepinephrine-induced preconstriction). Vasomotor function was not different between the sexes. More altered base pairs/variants were identified in Ossabaw than in Göttingen minipigs for the exon encoding adrenoceptor-α_1A_. Vasomotor function in lean Ossabaw minipigs is shifted toward vasoconstriction and away from vasodilation in comparison with Göttingen minipigs, suggesting a genetic predisposition for vascular dysfunction and atherosclerosis in Ossabaw minipigs. Thus, Ossabaw minipigs may be a better model for human cardiovascular disease than Göttingen minipigs.

**NEW & NOTEWORTHY** Animal models with a predisposition to metabolic syndrome and atherosclerosis are attracting growing interest for translational research, as they may better mimic the variability of patients with cardiovascular disease. In Ossabaw minipigs, with a polygenic predisposition to metabolic syndrome, but without the diseased phenotype, vasoconstriction is more and vasodilation is less pronounced in mesenteric arteries than in Göttingen minipigs. Ossabaw minipigs may be a more suitable model of human cardiovascular disease.

## Graphical abstract

**Figure:**
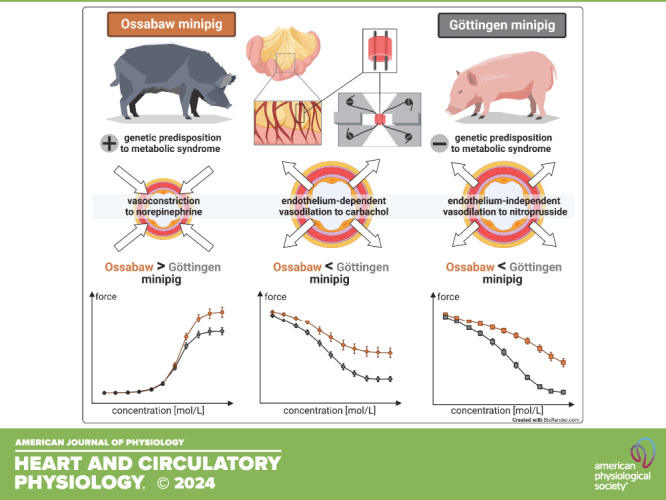

## INTRODUCTION

Atherosclerosis and subsequent cardiovascular disease are driven by a complex interaction of risk factors. Risk factors can be divided into nonmodifiable (genetic background, age, and sex) and modifiable risk factors (1, 2). Modifiable risk factors can be further subdivided into behavioral risk factors, such as nutrition/obesity, smoking, and physical inactivity, and those with a strong genetic background, such as dyslipidemia, hypertension, and insulin resistance/diabetes [the combination of the latter four is defined as metabolic syndrome) (3–6)]. Because the metabolic syndrome combines several risk factors (7, 8), it is a particularly high-risk condition. Primary prevention by treating modifiable risk factors provides significant benefits to patients by reducing the risk for the manifestation of atherosclerosis and cardiovascular diseases (9–12). Primary prevention, however, does not prevent the development of risk factors (11). Thus, the so-called primordial prevention has recently gained more attention (9–12). In recent decades, there has been great progress in the understanding of the underlying molecular and cellular mechanisms of atherosclerosis development, but the interaction between nonmodifiable, particularly the polygenic, and modifiable risk factors, is still not clear (4). Clear is, however, that such interaction of nonmodifiable and modifiable risk factors increases an individual’s risk of developing cardiovascular disease (4). Basic research in this field is challenging since translationally relevant risk factors are not readily reflected in most preclinical animal models. In established animal models (mouse, rat, rabbit, pig), the combination of a high-cholesterol diet and/or monogenic modifications is often used to establish vascular dysfunction and dyslipidemia-induced atherosclerotic plaque formation (13, 14). However, for the development of additional risk factors such as diabetes, further specific diets or pharmacological/surgical interventions are often required (14). Despite these efforts, the animal models do not reflect the complex human situation of a heterogeneous, polygenic determined primordial risk constellation for the development of metabolic syndrome, which predisposesvascular dysfunction and atherosclerotic cardiovascular disease (13–15).

The feral pig breed, the Ossabaw minipig, features a “thrifty genotype” as an adaptation to survive periods of low food availability (16). After consumption of a hypercaloric, atherogenic diet, Ossabaw minipigs develop full metabolic syndrome including obesity, glucose intolerance, insulin resistance, hypertension, and dyslipidemia. The syndrome leads to vascular dysfunction, and diffuse coronary atherosclerosis, including plaque instability and subsequent thrombosis on a polygenic background (17–20). Such a “thrifty genotype” probably exists also in humans and may explain a genetic predisposition to cardiovascular disease when associated with a Western lifestyle (21, 22).

Indeed, there are strain-specific differences in coronary microvascular function, as evidenced by reduced coronary flow reserve in response to adenosine and bradykinin in lean Ossabaw minipigs as compared with Yucatan minipigs (23). So far, differences in the coronary circulation with respect to α_1_- and α_2_-adrenergic vasoconstriction, which is absent in crossbreed landrace × Yorkshire pigs and Göttingen minipigs, but present in dogs and humans, have been considered to be species specific (24–29), but they may well also be strain specific. The genetically determined primordial risk constellation for the metabolic syndrome, as present in Ossabaw minipigs, but also in humans, may impact vascular function and reflect an early manifestation of atherosclerosis.

Thus, we now attempted to identify possible differences in the vascular function of lean Ossabaw minipigs, which still had a healthy phenotype and only the predisposition to metabolic syndrome, with that of Göttingen minipigs, an important minipig breed in coronary vascular disease research (19, 28). Unlike Ossabaw minipigs, Göttingen minipigs do not have such a polygenically determined risk for the development of metabolic syndrome. Göttingen minipigs were bred in the 1960s by crossing Minnesota minipigs, Vietnamese pot-bellied pigs, and the German landrace, and this strain has been under a fully documented, closed, selective breeding scheme ever since (30).

The process of atherosclerosis is a generalized process affecting all vascular beds, which has been suggested to start in resistance arteries before changes in conduit arteries (31, 32). We used mesenteric resistance arteries, as they are easily accessible and feature a high reproducibility in isometric force measurements (33, 34). For comparison of in vitro vascular function between these minipig strains, we used pigs undergoing cardioprotection studies (35–37), in compliance with the replacement, refinement, and reduction, the “3R”, of animals in research (38, 39).

## MATERIAL AND METHODS

The authors declare that all supporting data of the present study are available in the article. Experiments were performed between July, 2020 and October, 2022. The experimental protocols conform to the National Institutes of Health’s *Guide for the Care and Use of Laboratory Animals*, the ‘‘Position of the American Heart Association on Research Animal Use,’’ adopted on November 11, 1984, and the Animal Research: Reporting of In Vivo Experiments (ARRIVE) guidelines. The experimental protocols in pigs were approved by the Bioethical Committee of the district of Düsseldorf (G1610/17; G1777/20). Mesenteric arteries were isolated from contemporary Göttingen minipigs without (*n* = 17) or with (*n* = 23) prior ischemic preconditioning (IPC) undergoing sustained myocardial ischemia/reperfusion (37) and Ossabaw minipigs without (*n* = 18) or with (*n* = 12) IPC (35, 36), which have been reported before. We assumed that regional myocardial IPC has no systemic effects on mesenteric artery vasomotor function. We therefore pooled the data from minipigs with and without IPC before myocardial infarction, respectively. However, to confirm our assumption, we reanalyzed the vasomotor function data and stratified them without/with IPC. Pigs that had been not included in previous studies focusing on cardioprotection, e.g., due to surgical complications, problems during the intervention, an increased ischemic blood flow, or severe hemodynamic instability were used in this study [21 additional Göttingen minipigs (*n* = 9 without and *n* = 12 with IPC) and one additional Ossabaw minipig with IPC], assuming that the exclusion criteria did not affect the parameters studied here. As previously described (35–37), Göttingen minipigs [females, males, and castrated (at 4 wk of age) males; Ellegaard, Dalmose, Denmark] and Ossabaw minipigs [females and castrated (at 4 wk of age) males; CorVus Biomedical, Crawfordsville, Indiana] were fed with standard chow (Göttingen minipigs, 300 g twice/day; Ossabaw minipigs: 500 g twice/day; Ssniff, No. V4133, Soest, Germany). Pigs had access to water ad libitum and were kept in tiled rooms (∼2 m^2^/pig) with straw bedding at 12-h:12-h light/dark cycles. The phenotypic features of Ossabaw and Göttingen minipigs, including body weight, age, and serum glucose and lipids (blood drawn after induction of anesthesia) are shown in Table 1.

**Table 1.** Phenotypic features of female and castrated male Ossabaw minipigs and female, male, and castrated male Göttingen minipigs for mesenteric vasomotor assay

	Ossabaw Minipigs	Göttingen Minipigs	*P* Value
Minipigs, *n*			
Females	19	9	
Males	–	22	
Castrated males	11	9	
Age, mo	18 ± 2	15 ± 2	<0.05
Body weight, kg	55 ± 6	38 ± 8	<0.05
Temperature, °C	37 ± 3	38 ± 1	
Heart rate, beats/min	99 ± 10	108 ± 12	<0.05
Left ventricular pressure, mmHg	94 ± 11	84 ± 10	<0.05
Erythrocytes,10^6^/mm^3^	5.9 ± 0.7	5.8 ± 0.8	
Leukocytes,10^3^/mm^3^	7.7 ± 1.4	5.9 ± 1.3	<0.05
Platelets, 10^3^/mm^3^	262 ± 41	452 ± 88	<0.05
Na^+^, mmol/L	144 ± 6	142 ± 3	
K^+^, mmol/L	3.7 ± 0.3	4.1 ± 0.5	<0.05
Glucose, mg/dL	131 ± 56	100 ± 34	<0.05
Cholesterol, mg/dL	74 ± 11	62 ± 20	<0.05
HDL, mg/dL	31 ± 6	32 ± 9	
LDL, mg/dL	33 ± 7	22 ± 11	<0.05
Triglycerides, mg/dL	41 ± 14	44 ± 13	
hs-CRP, mg/dL	<0.02	<0.02	
AST, U/L	48 ± 10	37 ± 7	<0.05
GPT, U/L	81 ± 23	49 ± 9	<0.05
Creatinine, mg/dL	1.03 ± 0.17	0.77 ± 0.18	<0.05

Values are means ± SD. AST, aspartate aminotransferase; GPT, glutamate pyruvate transaminase; HDL, high-density lipoprotein; hs-CRP, high-sensitivity C-reactive protein; LDL, low-density lipoproteins. Unpaired Student’s *t* test.

Unless otherwise specified, materials were obtained from Sigma Aldrich (Deisenhofen, DE). All chemicals for the Krebs–Henseleit buffer were purchased from AppliChem (Darmstadt, DE).

### Preparation of Mesenteric Arteries

At the end of the respective study protocol and after the excision of the heart, the abdomen was opened and the intestine was exposed. The mesenteric vessels of the small intestine were excised along the intestinal loop over a length of 10 cm and a width of 4–5 cm. The removed tissue was stored overnight in 4°C Krebs–Henseleit buffer, containing (in mmol/L) 119 NaCl, 4.7 KCl, 2.5 CaCl_2_·2H_2_O, 1.17 MgSO_4_·7H_2_O, 25 NaHCO_3_, 1.18 KH_2_PO_4_, 0.027 EDTA, and 5.5 glucose. Second-order mesenteric arteries were carefully dissected at 4°C and placed into carbogenated (5% CO_2_-95% O_2_) Krebs–Henseleit buffer at room temperature. For vasomotor measurements, the vessels were cut into segments of 2 mm in length and mounted in an isometric small vessel myograph (Danish Myo Technology, Aarhus, DK).

### Vasomotor Assay

The vasomotor assay has been described in detail previously (40–42) and complies with the guidelines for the measurement of vascular function and structure in isolated arteries (33). Briefly, segments of mesenteric resistance arteries (43) were mounted on two stainless steel wires (40 µm in diameter), which were connected to a force transducer and a micrometer, respectively. Arteries were equilibrated in carbogenated Krebs–Henseleit buffer at 37°C before an automated normalization procedure was performed. This normalization is controlled from the interface using a standardized procedure according to the manufacturer’s protocol. The normalization uses an approximation of the lumen diameter (*d*100) that the mesenteric artery would have had in vivo, when relaxed and subjected to a transmural pressure of 100 mmHg, and the Laplace law for vessels with infinitely thin walls: P = 2 *T*/*d*, where P is the transmural pressure, *T* is the wall tension and *d* is the lumen diameter. The arteries were then adjusted to a lumen diameter of *d* = 0.9 × *d*100, where active force development (measured in mN) is assumed to be maximal. The active force was divided by twice the length of the mesenteric segment (AD converter: PowerLab8/30, software: LabChart6, ADInstruments GmbH, Spechbach, DE) (33, 40–42). The vessels were equilibrated for a further 30 min with frequent buffer changes. The baseline developed force of contraction was measured (33, 34). Vasoconstriction was then repeatedly induced by depolarization of the vascular smooth muscle cell membrane with potassium chloride (KCl twice 0.6 × 10^−1^ mol/L and twice 1.2 × 10^−1^ mol/L over 5 min each). Between KCl exposures, the vessels were washed with frequent buffer changes until baseline force was reached again. The maximal vasoconstrictor response to KCl (KCl_max_) was determined at 1.2 × 10^−1^ mol/L KCl.

Cumulative concentration-response curves were determined in response to 1 × 10^−9^–1 × 10^−4^ mol/L for norepinephrine and expressed as ΔmN (difference to baseline developed force of contraction) and as a percentage of KCl_max_. Endothelium-dependent and endothelium-independent vasodilation were measured in response to carbachol and nitroprusside (each 1 × 10^−9^–1 × 10^−4^ mol/L) after maximal preconstriction by norepinephrine and expressed as ΔmN (difference to baseline developed force of contraction) and as a percentage of norepinephrine-induced preconstriction.

### Bioinformatic Analysis of Differences between the Ossabaw Minipig and Göttingen Minipig Genomes

Previously, the Ossabaw minipig genome has been analyzed and compared with that of Göttingen minipigs using a k-mer-based method, and the genome of *Sus scrofa* as reference (35). Variants and altered base pairs (bp) within the sequences annotated for *Sus scrofa* were detected (*Sus scrofa* vs. Ossabaw minipig; *Sus scrofa* vs. Göttingen minipig). In the present study, we used these genome data (35) and focused on those genes, that have been associated with altered vasomotor function and coronary artery disease in patients, i.e., eNOS (44, 45) and protein-encoding exons involved in adrenoceptors-mediated vasomotor function (46–50). We additionally focused on genes identified by human genome-wide association studies (GWAS), which are statistically associated with coronary artery disease and myocardial infarction and divided into key pathological pathways (i.e., extracellular matrix/vascular remodeling, nitric oxide/cGMP and vasoreactivity/endothelin pathway, cholesterol/lipid metabolism, immune response/inflammation, TGF-β signaling pathway, angiogenesis/vascularization, cell cycle, and growth regulation) (51, 52).

### Statistics

Investigators analyzing vascular function were blinded with respect to sample origin (pig strain and study protocol). Continuous data are presented as means ± SD. Individual data of baseline diameter, baseline developed force of contraction, and KCl_max_ are presented in boxplots and as single data points. The single data points represent the average of one to six vessels per pig. Vasoconstriction to norepinephrine and endothelium-dependent and endothelium-independent vasodilation are presented as means ± SD. Means ± SD were calculated from averaged values of one to six vessels per individual pig.

Data were tested for normality using the Kolmogorov–Smirnov test. Unpaired Student’s *t* test was used to analyze *1*) phenotypic features, *2*) baseline diameter, *3*) baseline developed force of contraction, and *4*) KCl_max_. Two-way analysis of variance (pig strain/concentration of the vasoconstrictor/vasodilator) for repeated measures was used to compare *1*) vasoconstriction in response to norepinephrine, *2*) endothelium-dependent vasodilation in response to carbachol, and *3*) endothelium-independent vasodilation in response to nitroprusside. When a significant difference was detected, individual mean values were compared by Fisher’s least significant post hoc test. Differences were considered significant at the level of *P* value < 0.05.

Unpaired Student’s *t* test was performed using Microsoft Excel 2016 software (Microsoft, Redmond, WA). The Kolmogorov–Smirnov test and the two-way analysis of variance for repeated measures were performed with SigmaStat (SigmaStat 3.5; SPSS Chicago, IL).

## RESULTS

### Stronger Vasoconstriction and Lesser Endothelium-Dependent and Endothelium-Independent Vasodilation in Mesenteric Arteries of Ossabaw Minipigs than Göttingen Minipigs

**Figure 1.**
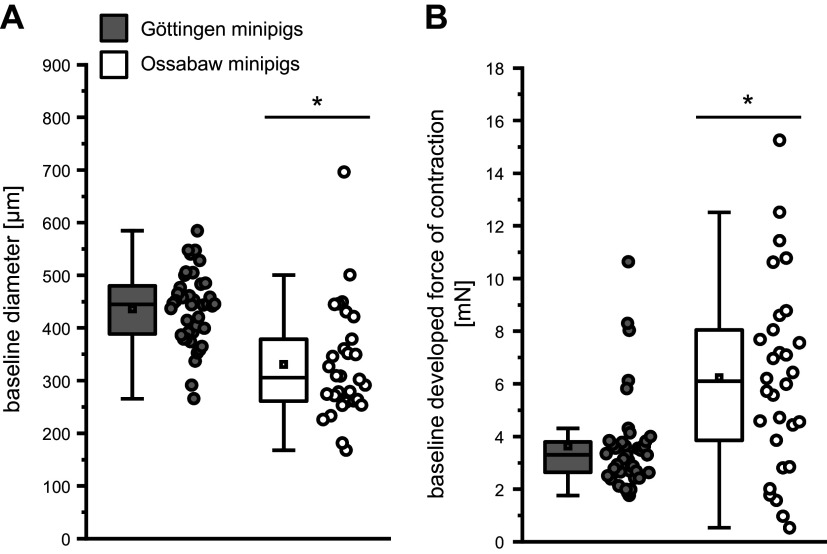
Smaller baseline vessel diameter (*A*) and higher baseline developed force of contraction (*B*) in mesenteric arteries of Ossabaw than of Göttingen minipigs. Data are presented as boxplots with single data points [minimum and maximum (whiskers), interquartile ranges from 25 to 75% (box), and means (square)]. Single data points represent the averages of 1–6 vessels per pig, respectively; 184 vessels from *n* = 40 Göttingen minipigs, and 118 vessels from *n* = 30 Ossabaw minipigs; unpaired Student’s *t* test: *P < 0.05 vs. Göttingen minipigs.

**Figure 2.**
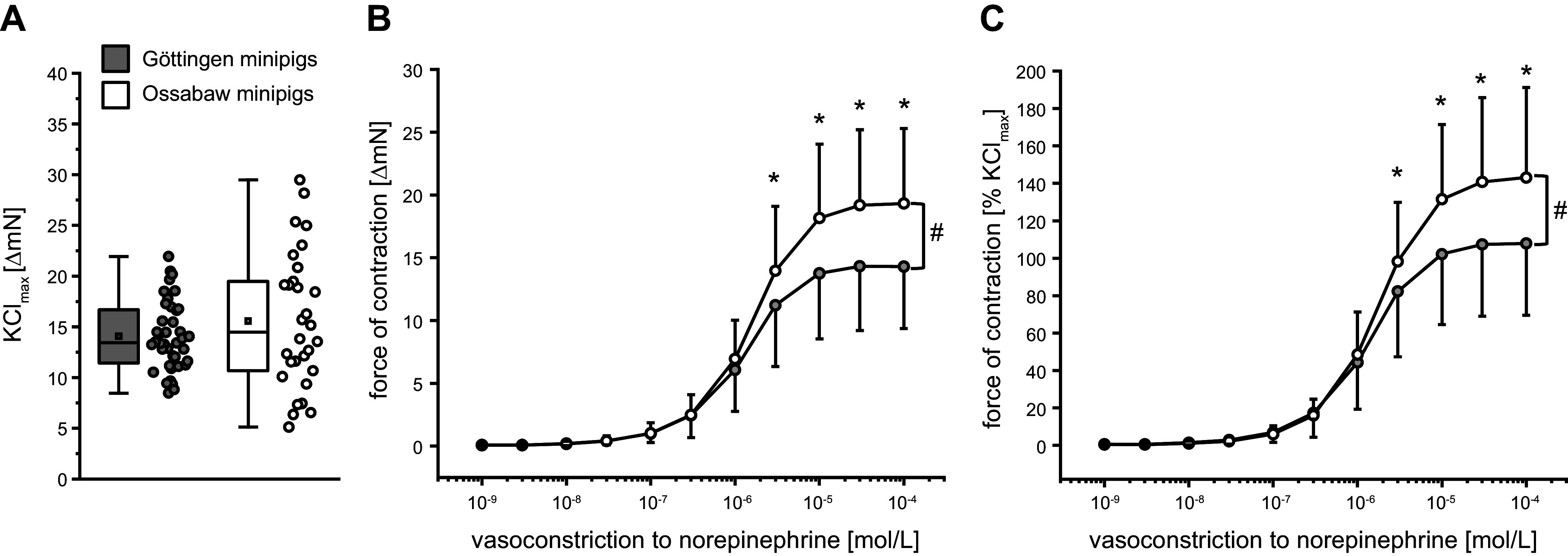
*A*: comparable maximal vasoconstriction to potassium chloride (KCl_max_) in mesenteric arteries of Göttingen and Ossabaw minipigs. *B* and *C*: vasoconstriction in response to norepinephrine was more pronounced in vessels of Ossabaw than of Göttingen minipigs. Data are presented as boxplots with single data points [minimum and maximum (whiskers), interquartile range from 25 to 75% (box), means (square), and median (line) (*A*)] and as means ± SD (*B* and *C*). The means ± SD were calculated from averages of 1–6 vessels per pig, respectively; 184 vessels from *n* = 40 Göttingen minipigs and 118 vessels from *n* = 30 Ossabaw minipigs: unpaired Student’s *t* test: *P* = 0.23 Göttingen minipigs vs. Ossabaw minipigs (*A*); and two-way analysis of variance for repeated measures: #*P* < 0.05 Göttingen minipigs vs. Ossabaw minipigs, Fisher’s least significant difference post hoc test (*B* and *C*). *P < 0.05 vs. Göttingen minipigs.

The baseline diameter of mesenteric arteries from Ossabaw minipigs was smaller than that from Göttingen minipigs (329 ± 108 vs. 435 ± 69 µm; Fig. 1*A*), and mesenteric arteries of Ossabaw minipigs developed a higher baseline force of contraction than mesenteric arteries of Göttingen minipigs (6.2 ± 3.6 vs. 3.6 ± 1.8 mN; Fig. 1*B*). KCl_max_ was similar between the mesenteric arteries of Ossabaw minipigs and Göttingen minipigs (15.6 ± 6.7 vs. 14.1 ± 3.4 ΔmN; Fig. 2*A*). The force of contraction in response to norepinephrine was more pronounced in mesenteric arteries of Ossabaw than Göttingen minipigs (1 × 10^−4^ mol/L norepinephrine: increase of force to 19.3 ± 6.0 vs. 14.3 ± 4.9 ΔmN, see Fig. 2*B*; increase of force to 143 ± 48 vs. 108 ± 38% of KCl_max_, see Fig. 2*C*).

**Figure 3.**
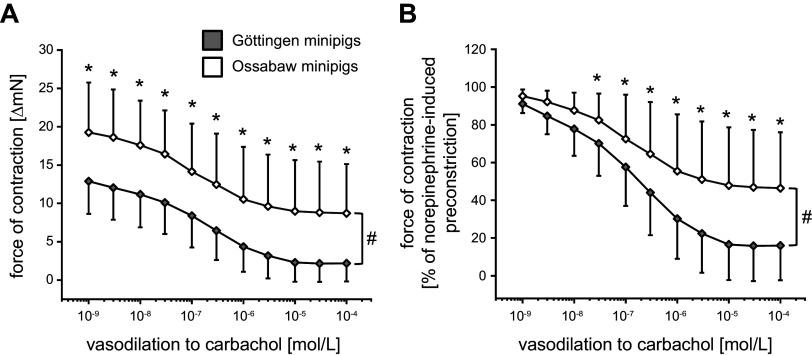
Endothelium-dependent vasodilation to carbachol was less pronounced in mesenteric arteries of Ossabaw minipigs than of Göttingen minipigs. Data are presented as means ± SD. The means ± SD of means were calculated from averages of 1–3 vessels per pig, respectively; 97 vessels of *n* = 37 Göttingen minipigs and 60 vessels of *n* = 29 Ossabaw minipigs. *A* and *B*: two-way analysis of variance for repeated measures: #*P* < 0.001 Göttingen minipigs vs. Ossabaw minipigs, Fisher’s least significant difference post hoc test. *P < 0.05 vs. Göttingen minipigs.

**Figure 4.**
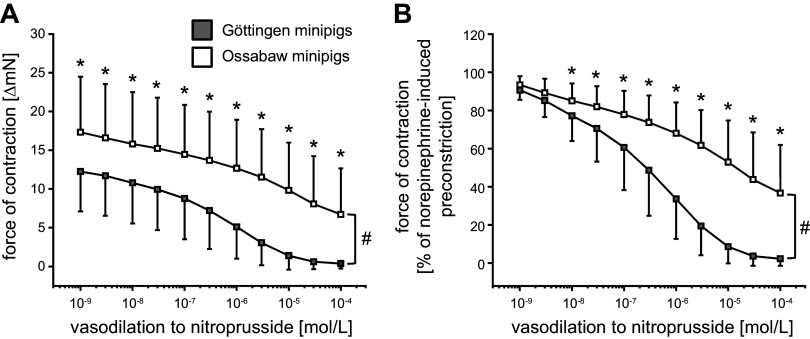
Endothelium-independent vasodilation to nitroprusside was less pronounced in mesenteric arteries of Ossabaw minipigs than of Göttingen minipigs. Data are presented as means ± SD. The means ± SD were calculated from averages of 1–3 vessels per pig, respectively; 87 vessels of *n* = 38 Göttingen minipigs and 58 vessels of *n* = 27 Ossabaw minipigs. *A* and *B*: two-way analysis of variance for repeated measures: #*P* < 0.001 Göttingen minipigs vs. Ossabaw minipigs, Fisher’s least significant difference post hoc test. *P < 0.05 vs. Göttingen minipigs.

Endothelium-dependent vasodilation was less pronounced in mesenteric arteries from Ossabaw than from Göttingen minipigs (1 × 10^−4^ mol/L carbachol: decrease of force to 8.7 ± 6.4 vs. 2.2 ± 2.4 ΔmN, see Fig. 3*A*; decrease of force to 46.4 ± 29.6 vs. 16.0 ± 18.4% of norepinephrine-induced preconstriction, see Fig. 3*B*). Endothelium-independent vasodilation was also less pronounced in mesenteric arteries from Ossabaw minipigs than from Göttingen minipigs (1 × 10^−4^ mol/L nitroprusside: decrease of force to 6.7 ± 5.9 vs. 0.4 ± 0.6 ΔmN, see Fig. 4*A*; decrease of force to 36.7 ± 25.2 vs. 2.3 ± 3.7% of norepinephrine-induced preconstriction, see Fig. 4*B*).

The differences in baseline diameter, in baseline developed force of contraction, as well as preconstriction to norepinephrine between the mesenteric arteries from Ossabaw minipigs and Göttingen minipigs may have affected the magnitude of vasoconstriction and vasodilation. Therefore, we retrospectively matched subsets of mesenteric arteries with a comparable baseline diameter (Supplemental Fig. S1; Supplemental data may be found at https://doi.org/10.6084/m9.figshare.24558541.v2), a comparable baseline developed force of contraction (Supplemental Fig. S2), or a comparable preconstriction with norepinephrine (10^−4^ mol/L; Supplemental Fig. S3) and reanalyzed their vasomotor function. Neither the baseline vessel diameter nor the baseline developed force of contraction nor the preconstriction to norepinephrine had any effect on the abovementioned vasomotor function data (Supplemental Figs. S1–S3).

Sex had no effect on vasomotor function in either Ossabaw or Göttingen minipigs (Supplemental Fig. S4). The cardioprotective maneuver, IPC, also did not affect vasomotor function (Supplemental Fig. S5).

### Altered Base Pairs and Variants in Protein Encoding Genes Involved in Vasoconstriction and Vasodilation and/or Associated with Coronary Artery Disease and Myocardial Infarction in Ossabaw and Göttingen Minipigs

The exon encoding adrenoceptor-α_1A_ had by comparison with *Sus scrofa* several thousand altered bp/Mbp and variants/Mbp in Ossabaw minipigs, but lesser altered bp/Mbp and variants/Mbp in Göttingen minipigs. Exons of the other selected proteins had by comparison with *Sus scrofa* few or no altered bp/Mbp and variants/Mbp in Ossabaw minipigs but, several thousand altered bp/Mbp and variants/Mbp in Göttingen minipigs (Table 2).

**Table 2.** Excerpt of supplemental data file

		Ossabaw Minipig		Göttingen Minipig	
Annotated Sequence	Sequence Length	Altered bp/Mbp	Variants/Mbp	Altered bp/Mbp	Variants/Mbp
α_1_-Adrenoceptor					
ADRA1A	**17,143**	**31,383**	**4,958**	**5,483**	**1,167**
ADRA1B	3,280	305	305	230,488	3,354
ADRA1D	1,713	0	0	674,839	3,503
α_2_-Adrenoceptor					
ADRA2A	1,353	0	0	294,161	8,130
ADRA2B	1,341	0	0	68,606	9,694
ADRA2C	2,038	0	0	869,480	3,435
β-Adrenoceptor					
ADRB1	1,410	0	0	807,092	2,837
ADRB2	2,688	0	0	154,390	2,604
ADRB3	3,333	0	0	318,032	2,700
Cholinergic receptor muscarinic 3					
CHRM3	25,105	438	279	8,086	956
Nitric oxide synthase					
NOS1	11,467	0	0	33,226	3,052
NOS2	6,013	4,324	998	69,849	2,994
NOS3	4,016	0	0	547,809	8,964
Gq protein					
GNA11	1,083	0	0	128,347	3,693
GNA12	6,478	309	309	198,827	3,550
GNA13	6,309	0	0	87,177	793
GNA14	2,209	0	453	21,277	1,811
GNA15	6,141	0	0	71,812	4,397
GNB1	3,123	0	0	152,097	3,842
GNB2	2,651	0	0	102,980	5,658
GNB3	3,710	0	0	354,178	6,469
GNB4	5,827	0	0	39,986	1,201
GNB5	4,009	0	0	92,043	2,744

Altered base pairs and variants in Ossabaw minipigs and Göttingen minipigs, published in Kleinbongard et al. (35). Altered base pairs (bp) and variants in Ossabaw minipigs and Göttingen minipigs for the proteins, which are potentially involved in vasoconstriction to norepinephrine and endothelium-dependent and -independent vasodilation. Protein-encoding genes with a higher number of altered bp and/or variants in Ossabaw minipigs than in Göttingen minipigs where shown in boldface.

Among those genes, identified by human GWAS to be statistically associated with coronary artery disease and/or myocardial infarction (51, 52), the exons encoding “ATP binding cassette subfamily G member 5 (ABCG5),” “four and a half LIM domains 5 (FHL5),” “fibronectin 1 (FN1),” “guanylate cyclase-1 soluble subunit-α_1_ (GUCY1A3),” “histone deacetylase 9 (HDAC9),” “HDGF like 1 (HDGFL1),” “phosphotyrosine interaction domain containing 1 (PID1),” “plasminogen (PLG),” “RE1 silencing transcription factor (REST),” “semaphorin 5 A (SEMA5A),” and “Sushi von Willebrand factor type A, EGF, and pentraxin domain containing 1 (SVEP1)” had by comparison with *Sus scrofa* several thousand altered bp/Mbp and/or variants/Mbp in Ossabaw minipigs, but lesser altered bp/Mbp and/or variants/Mbp in Göttingen minipigs (Supplemental Table S1). Exons of the other reported proteins had by comparison with *Sus scrofa* few or no altered bp/Mbp and variants/Mbp in Ossabaw minipigs but, several thousand altered bp/Mbp and variants/Mbp in Göttingen minipigs.

## DISCUSSION

Ossabaw minipigs with a polygenic predisposition for the development of the metabolic syndrome, but without the diseased phenotype had smaller vessel diameter and higher baseline developed force of contraction in mesenteric arteries than Göttingen minipigs, without such predisposition. These differences suggest a higher active tension of the vascular smooth muscle cells at baseline (34, 53) of the Ossabaw than Göttingen minipig arteries. Ossabaw minipig arteries were further characterized by more pronounced vasoconstriction to norepinephrine and less pronounced endothelium-dependent and endothelium-independent vasodilation than those from Göttingen minipigs. This again suggests that Ossabaw minipig arteries have a higher contractile state and thus higher vascular resistance (33, 34). The lesser vasodilation to both, endogenous (endothelium-dependent) and exogenous (endothelium-independent) nitric oxide in Ossabaw than in Göttingen minipigs may indicate that endothelial dysfunction does not contribute quantitatively to the observed differences in vasomotor function between the two pig strains. Overall, the differences in vasomotor function were more pronounced when the vessel diameter was small/constricted, indicating a higher sensitivity to vasomotor changes in this condition. These in vitro differences were reflected in vivo by a somewhat elevated (∼10 mmHg, see Table 1) but not yet pathological blood pressure in Ossabaw minipigs compared with Göttingen minipigs. In a bioinformatic analysis, we identified a higher number of altered base pairs and variants for the protein encoding the adrenoceptor-α_1A_ in Ossabaw minipigs than in Göttingen minipigs, possibly supporting the differences in adrenergic vasoconstriction at the genetic level.

Our findings in mesenteric arteries extend those from previous studies on vascular function in coronary arteries of Ossabaw minipigs. When compared with Yucatan (23) and domestic pigs (54), pig strains without a genetic predisposition to metabolic syndrome and atherosclerosis, coronary arteries of Ossabaw minipigs were characterized by an impaired flow reserve in vivo associated with an altered Ca^2+^ homeostasis in vitro (23), a sustained histamine-induced vasoconstriction and an altered K^+^ homeostasis in vitro (54), reflecting endothelial dysfunction and a higher risk of coronary vasoconstriction.

Our data, in combination with such previously published data, suggest that genetically determined differences in vascular function in the Ossabaw minipigs appear to be the substrate for the increased risk of developing diffuse coronary atherosclerosis with plaque instability, as typical for the Ossabaw minipigs on a hypercaloric, atherogenic diet with fully developed metabolic syndrome. Ultimately, both the increased plaque burden (17, 19, 20, 23) and the increased vasoconstrictor responsiveness (23) of the poststenotic coronary microcirculation during sympathetic activation (25, 27, 29) may be responsible for the initiation of acute myocardial infarction in Ossabaw minipigs (17, 19, 20). Indeed, such scenarios closely mirror the situation in patients (25, 55). Vasomotor function data from Ossabaw minipigs displayed a considerably higher heterogeneity than those from Göttingen minipigs; this may be due to the higher genetic heterogeneity of the nonselective feral breed of Ossabaw minipigs (20) than the more selective breed of Göttingen minipigs (30).

Increased ischemic burden may be the result of increased α_1_- and α_2_-adrenergic coronary vasoconstriction (25, 27, 29), which has been observed in dogs (56) and humans (25, 57), but not in crossbred landrace pigs × Yorkshire pigs (26) and Göttingen minipigs (24, 25, 27–29). Although the affinity of norepinephrine for α-adrenoceptors is higher than that for β-adrenoceptors (58), differences in the balance between α-adrenergic vasoconstriction and β-adrenergic vasodilation may have influenced vascular reactivity to adrenergic stimulation (50). During the development of atherosclerosis, however, there is a shift toward more α-vasoconstriction (46, 48, 50). In the present study, Ossabaw minipigs arteries had a more pronounced adrenergic vasoconstriction than those of Göttingen minipigs, thus better mimicking the α-adrenergic-induced vasoconstriction as described in patients. However, the receptor arrangement (59–64) and consequently the vasomotor function (41, 65) of coronary and mesenteric arteries are only partially comparable. Therefore, future approaches using specific α_1_- and α_2_-adrenergic, as well as β-adrenergic receptor agonists and antagonists in coronary rather than mesenteric arteries are needed to elucidate possible species- and/or race-specific differences in adrenergic vasoconstriction. Stronger coronary α-adrenoceptor-mediated vasoconstriction in patients with unclear chest pain but without significant stenosis was associated with single nucleotide polymorphisms in, i.e., eNOS (44, 45) and G protein-coding exons (47, 49). To possibly associate these data with our previous findings on the Ossabaw minipigs, we used our previous DNA sequencing data from Ossabaw minipigs and the bioinformatic comparison using the k-mer-based method with the genomes of *Sus scrofa* and Göttingen minipigs (35). Indeed, the exon encoding the α-adrenoceptor 1A had more altered base pairs/variants in Ossabaw minipigs than in Göttingen minipigs. Among those genes associated with coronary artery disease and myocardial infarction in humans (51, 52) and those with more altered base pairs/variants in Ossabaw than in Göttingen minipigs, two, namely, FHL5 and GUCY1A3, are associated with vasomotor function. FHL5 promotes a contractile smooth muscle cell phenotype through dysregulation of vascular remodeling and calcium handling (66), GUCY1A3 is a key enzyme in the nitric oxide/cAMP signaling pathway (67) and is thus involved in smooth muscle cell relaxation. However, for all other possible relevant proteins, we could not identify differences in protein-encoding genes that might explain the differences in vasomotor function between Ossabaw and Göttingen minipigs. In our previous study, however, we have identified differences in protein-coding genes between Ossabaw and Göttingen minipigs in fat metabolism, mitochondria, and inflammation [including the Janus kinase (JAK)-signal transducer and activator of transcription (STAT) pathway] (35). Alterations in lipid metabolism (4), as well as mitochondrial dysfunction in endothelial and vascular smooth muscle cells (68), are important drivers for the initiation and progression of atherosclerosis. Also, the JAK-STAT pathway regulates the initiation and progression of atherosclerosis by facilitating vascular cell inflammation, proliferation, and migration (69). All these comparisons, however, used the annotated genome *Sus scrofa* reference, because neither the Ossabaw nor the Göttingen minipig genomes are annotated (35), and are therefore only indirect and rough. Also, the k-mer-based method, which analyzes variants in the exons of protein-coding genes, does not permit comprehensive conclusions on gene polymorphisms associated with altered vasomotor function and coronary artery disease (44–52), nor does it provide information on protein expression or even protein activity.

Genetic differences between pig strains affect not only vasomotor function but also the cardiovascular responses to pharmacological agents, i.e., the β-blocker metoprolol. Both the effect of metoprolol on heart rate (70) and the heart rate-independent reduction in infarct size by metoprolol (70–74) differed between pig breeds [Yorkshire pigs (71), Large White pigs (72, 73), and Göttingen minipigs (74)].

Importantly, it remains open whether the differences found in the vasomotor function and the genome data-based analysis reflect the genetic distance of the Göttingen minipigs to feral or other pig breeds, as described in the study by Reimer et al. (30), or reflect a pathological predisposition of Ossabaw minipigs to atherosclerosis and ischemia.

In conclusion, Ossabaw minipigs with a primordial predisposition to metabolic syndrome and atherosclerosis may better mimic the human situation and thus be of greater interest for translational approaches to cardiovascular research than Göttingen minipigs (7, 14, 75–77); their altered vasomotor function, even before the pathological phenotype, may be the first evidence of early atherosclerosis in these pigs (78).

### Limitations

In terms of replacement, refinement, and reduction, the “3R”, of animals in research (38, 39), we used animals undergoing cardioprotective studies (35–37); this resulted in several limitations. Removed tissue was stored overnight at 4°C; however, Sams et al. (65) showed that a 24-h storage time did not alter the in vitro vasomotor function, and these conditions were the same for both pig strains. The animals underwent open-chest surgery with/without the cardioprotective intervention IPC before tissue harvesting. The cardioprotective intervention IPC, which had an infarct size-reducing effect in Göttingen minipigs, but not in Ossabaw minipigs (35, 37, 77), however, did not affect the in vitro vasomotor function measurements (see Supplemental Fig. S4). We did not identify sex-specific differences in vasomotor function, as described in humans and rats (79, 80). We used adult but still young pigs, and sex differences in vasomotor function result not only from genetic differences between the sexes but also from sex- and age-related changes in hormones (81, 82). We determined the vessel diameter in the presence of calcium; the use of a calcium-free buffer would have allowed the determination of the vessel diameter in a completely relaxed state (34). We used only norepinephrine as a vasoconstrictor and only one endothelium-dependent and one endothelium-independent vasodilator. The use of further vasoconstrictor and vasodilator agents as well as endothelial-denuded vessels would have characterized vasomotor function in more detail (33).

## DATA AVAILABILITY

Data will be made available upon reasonable request.

## SUPPLEMENTAL DATA

10.6084/m9.figshare.24558541.v2 Supplemental Table S1 and Supplemental Figs. S1–S5: https://doi.org/10.6084/m9.figshare.24558541.v2.

## GRANTS

This work was supported by German Research Foundation Grant SFB 1116 B08 (to G.H. and P.K.) and European Cost Action in Cardioprotection Grants CA16225 (to G.H.) and IG16225 (to G.H. and P.K.).

## DISCLOSURES

Michael Sturek is cofounder and Chief Scientific Officer of CorVus Biomedical, LLC, which produces Ossabaw minipigs. None of the other authors has any conflicts of interest, financial or otherwise, to disclose.

Petra Kleinbongard is an editor of *American Journal of Physiology-Heart and Circulatory Physiology* and was not involved and did not have access to information regarding the peer-review process or final disposition of this article. An alternate editor oversaw the peer-review and decision-making process for this article.

## AUTHOR CONTRIBUTIONS

P.K. conceived and designed research; C.E., H.R.L., and P.K. performed experiments; C.E. and P.K. analyzed data; C.E., M.S., G.H., and P.K. interpreted results of experiments; C.E. prepared figures; C.E. drafted manuscript; M.S., G.H., and P.K. edited and revised manuscript; C.E., H.R.L., M.S., G.H., and P.K. approved final version of manuscript.
